# How much does effective health facility inspection cost? An analysis of the economic costs of Kenya’s Joint Health Inspection innovations

**DOI:** 10.1186/s12913-022-08727-3

**Published:** 2022-11-14

**Authors:** Timothy Chege, Francis Wafula, Eric Tama, Irene Khayoni, Dosila Ogira, Njeri Gitau, Catherine Goodman

**Affiliations:** 1 Institute of Healthcare Management, Strathmore University, 59857, Ole Sangale Road, 00200 Nairobi, Kenya; 2 International Finance Corporation, Work Bank Group, 30577, Upper Hill Road, 00100 Nairobi, Kenya; 3 Department of Global Health and Development, London School of Hygiene and Tropical Medicine, Keppel, WC1E 7HT London, UK

**Keywords:** Regulation, Cost analysis, Digital innovation

## Abstract

**Background:**

In most low- and middle-income countries, health facility regulation is fragmented, ineffective and under-resourced. The Kenyan Government piloted an innovative regulatory regime involving Joint Health Inspections (JHI) which synthesized requirements across multiple regulatory agencies; increased inspection frequency; digitized inspection tools; and introduced public display of regulatory results. The pilot significantly improved regulatory compliance. We calculated the costs of the development and implementation of the JHI pilot and modelled the costs of national scale-up in Kenya.

**Methods:**

We calculated the economic costs of three phases: JHI checklist development, start-up activities, and first year of implementation, from the providers’ perspective in three pilot counties. Data collection involved extraction from expenditure records and key informant interviews. The annualized costs of JHI were calculated by adding annualized development and start-up costs to annual implementation costs. National level scale-up costs were also modelled and compared to those of current standard inspections.

**Results:**

The total economic cost of the JHI pilot was USD 1,125,600 (2017 USD), with the development phase accounting for 19%, start-up 43% and the first year of implementation 38%. The annualized economic cost was USD 519,287, equivalent to USD 206 per health facility visit and USD 311 per inspection completed. Scale up to the national level, while replacing international advisors with local staff, was estimated to cost approximately USD 4,823,728, equivalent to USD 103 per health facility visit and USD 155 per inspection completed. This compares to an estimated USD 86,997 per year (USD 113 per inspection completed) spent on a limited number of inspections prior to JHI.

**Conclusion:**

Information on costs is essential to consider affordability and value for money of regulatory interventions. This is the first study we are aware of costing health facility inspections in sub-Saharan Africa. It has informed debates on appropriate inspection design and potential efficiency gains. It will also serve as an important benchmark for future studies, and a key input into cost-effectiveness analyses.

**Supplementary Information:**

The online version contains supplementary material available at 10.1186/s12913-022-08727-3.

## Background

Achieving universal health coverage goes beyond expanding access. Increased access to good quality care, and a high degree of patient safety, are vital for improved outcomes [1]. In low- and middle-income countries (LMICs), lack of access to safe medical care contributes to about 25.9 million adverse events each year [2], with poor quality causing 5.7 to 8.4 million deaths [3]. Such inadequacies have focused attention on the role of government regulation in enforcing minimum standards of patient safety. Healthcare regulation is considered a key government stewardship function, defined as purposive actions initiated, although not necessarily implemented, by Government to address failures in the existing public and private health care system and promote current policy objectives [4]. However, the pluralistic and highly fragmented nature of LMIC health systems poses a major challenge for effective regulation [5]. Moreover, healthcare regulation in LMICs is often fragmented, ineffective, and poorly coordinated across agencies, which are frequently severely under-resourced [6]. Enhanced regulatory systems and enforcement is required in LMIC settings, to ensure effective stewardship. However, the cost of implementing effective regulation and its cost-effectiveness is a concern to policymakers, faced with numerous competing demands on limited national resources.

In Kenya, the Ministry of Health (MoH) and health regulatory agencies have piloted an innovative regulatory regime for health facilities involving Joint Health Inspections (JHI). In the past, each of the eight main regulatory agencies for doctors and dentists, clinical officers, nurses, public health officers, pharmacies, laboratories, radiologists and nutrition and dieticians had their own regulatory requirements for facilities. Inspections were only conducted in private facilities, and were sporadic and very patchy, covering only two to three regions and less than 5% of private health facilities annually. The JHI combined the requirements across all agencies to provide a common inspection framework. All public and private facilities received increased frequency of inspection using a comprehensive Joint Health Inspections Checklist (JHIC), with a target of 100% of public and private facilities inspected at least once per year. The JHIC is a regulatory tool, mainly focused on the minimum structural (input) indicators required to provide good quality care [7]. The tool checks compliance with minimum staff requirements (qualification and licensing), facility infrastructure, supplies and utilities, and professionally defined standards for specific service areas and units (e.g., theatre, labor ward, laboratory and pharmacy). There are also a limited number of process quality indicators, such as evidence of handwashing, monitoring labor and safe disposal of waste.

Joint Health Inspections were conducted by trained inspectors representing all regulatory bodies. Inspection data were entered on an electronic JHIC tool using tablets, which auto-generated inspection scores and reports and transferred results to an online management information system. The inspection protocols incorporated insights from risk-based and responsive regulatory theories [8, 9]. Facilities were risk-rated using a composite score based on inspection performance, with warnings, sanctions and time to re-inspection depending on these scores. Facilities outside the lowest compliance category were not penalized for infringements on their first inspection, but informed about their performance, with closure only to be implemented if sufficient improvements had not taken place at later inspection. In a sub-group of facilities letter symbol scorecards were publicly displayed outside facilities showing their JHIC score. Implementation and results of inspections were recorded on an online monitoring system.

The JHI was piloted in three of Kenya’s 47 counties in 2017 [10]. The pilot was evaluated under the Kenya Patient Safety Impact Evaluation (KePSIE) using a randomized controlled trial (RCT) study design with the support of the World Bank Group. This was the first RCT to look at the impact of regulations or inspections in health facilities. After one year of implementation, JHI inspection scores were 15% higher in treatment facilities compared to the control group (41% vs. 35% compliance), with larger improvements in private facilities (19% in private vs. 7% in public) [11]. This may have reflected higher baseline scores in public facilities (24% higher than in private sector), meaning there was more room for improvement in private facilities, which also received on average a higher number of inspection visits. There was no significant effect from adding public display of inspection results on scorecards. Overall, these improvements saw 19% of the facilities move from the “minimally compliant” category to higher compliance categories.

Following the successful JHI pilot, several other African countries have expressed interest in the JHI model, and the Kenyan Government is in the process of adapting and scaling-up the reforms nationwide. A key scale-up challenge is the sheer number of facilities to be inspected, and the associated resource implications. During the JHI pilot, around 856 facilities were inspected across three counties; the scale-up will involve over 12,000 facilities [12].

Costs of such health systems interventions are rarely assessed rigorously. In fact, we were not able to identify any published evidence on the cost and cost-effectiveness of health facility regulation and inspection in LMICs. However, information on the costs of these enhanced inspections is urgently needed to consider their affordability and value for money. This study aimed to calculate the economic costs of the development and implementation of the JHI pilot in the three counties, and to model the costs of national scale-up in Kenya.

## Methods

### The Joint Health Inspections pilot and evaluation

JHI was piloted in public and private (for-profit and not for-profit) health facilities in the three counties of Kakamega, Kilifi, and Meru. These health facilities comprised mainly level 2 dispensaries run by clinical officers with no inpatient services and level 3 health centers also run by clinical officers, but which may admit patients, together with a smaller number of level 4 hospitals which have fully qualified doctors and inpatient wards, and one level 5 referral hospital per county. The three counties were selected due to their wide diversity of market sizes (number and distribution of health facilities), and to ensure a broadly representative mix of regions within the country (Table 1). Across the three counties, the health facilities served over 4.5 million people, and received around 7 million patient visits per year [10].

**Table 1:** Characteristics of jhi pilot study counties

	**Kakamega**	**Kilifi**	**Meru**	**National**	**Source**
Region	Western	Coast (South)	Central/Eastern	N/A	
County size (Square km)	3,051	12,609	6936	581,313	
Population (Number of people)	1,867,579	1,453,787	1,545,714	47,564,296	[13]
Population density (Per square km)	612	115	223	82	
Number of health facilities (as of May 2019)	322	345	533	12,438	[14]

Advisory and governance support for the pilot was provided by a World Bank core team and the KePSIE Task Force comprising national and county health officials, managers of the eight regulatory agencies, and private sector representatives. On average 8 full-time inspectors were working in the selected counties, seconded from the national regulatory agencies, with one vehicle provided for each county. Inspectors were supported by two World Bank field staff in each county, who also oversaw the KePSIE evaluation, with logistics and management support from the Kenya Medical Practitioners and Dentists Council (KMPDC). An MoH coordinator was responsible for managing and supervising implementation and for conducting closures, supported by the host county health management team.

The JHIC categorized facilities into compliance categories based on their inspection score (Table 2). Facilities without a valid license for their facility or key departments (e.g., laboratory, pharmacy) were given a 90-day grace period to rectify this, after which they would be referred for closure, together with any rare cases of facilities scoring below 10% on the JHIC score. Facilities outside this lowest “non-compliant” category were not penalized for infringements on their first inspection, but time to reinspection was positively related to compliance category. Facilities in the “minimally” and “partially” compliant categories were to be closed if they had not made sufficient improvements by the third inspection visit, though in practice few facilities reached this stage during the one year of pilot implementation.

**Table 2:**
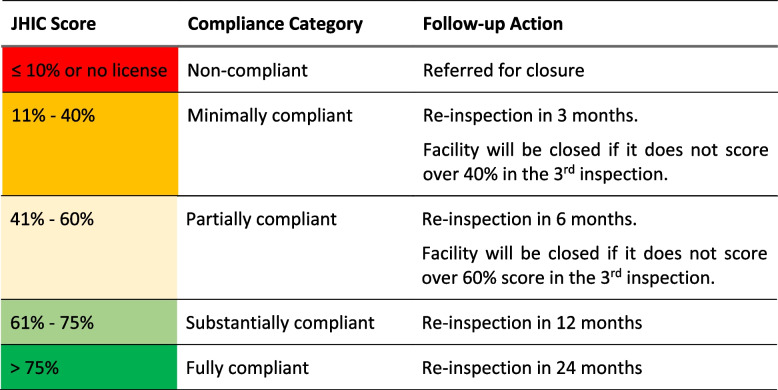
Joint Health Inspection Checklist (JHIC) scores and follow-up actions

We classified JHI activities into three phases for costing purposes: development phase (October 2013 to December 2014), start-up phase (January 2015 to November 2016), and implementation phase (November 2016 to December 2017). The activities under each phase are summarized in Table 3.

**Table 3:** Description of JHI pilot activities by phase

**Activities**	**Description of tasks**
**Development phase (October 2013 to December 2014)**	
Development of JHIC	JHIC developed by a Technical Working Group based on views of a wide range of healthcare regulation stakeholders through a series of meetings. World Bank team tested draft checklist in 42 health facilities in Nairobi and simulated results for a range of scoring systems to inform the Technical Working Group discussions
Consensus building	Two KePSIE Task Force (KTF) meetings with representatives from World Bank to agree on the JHIC design and content
JHIC Gazettement	JHIC gazetted (official public communication of statutory change) under Legislative Supplement No. 25 as part of Legal Notice No. 46 in the Public Health Act (Cap. 22) on March 21, 2016, to be applied at the national level. This process was overseen by a legal counsel. The final gazettement date was delayed until 2016 but the cost of the legal counsel for this activity was included in the development phase as it was categorized as a development activity
**Start-up phase (January 2015 and November 2016)**	
Implementation preparation	National launch followed by Kickoff meetings in the three pilot counties. Sub-county representatives and inspectors distributed copies of JHIC to county health management teams (CHMTs) and health facility managers
Development of implementation manual	Manual prepared by World Bank staff and consultants contracted by World Bank to cover all details of pilot implementation
Training & recruitment of inspectors	25-day training of 8-member Inspector Training Expert Group (ITEG) representing all regulatory agencies. ITEG then trained 20 potential inspectors for 20 days (only 8 of these were subsequently involved in inspection at any one time)
Facility mapping	A census and mapping of all health facilities in each pilot county conducted, to supplement official government facility list
Preparation of materials	Contracts to private firms for: scorecard design and piloting through focus group discussions with general public in Nairobi County; SMS verification for checking authenticity of scorecards; printing of patient information leaflets
Development of e-JHIC	JHIC customized and converted into an electronic version to be used on tablets. The pilot e-JHIC was developed by the World Bank and had over 40 rounds of revision during the one-year pilot to apply updates to inspection protocols and guidelines. The revision to the e-JHIC continued into the implementation year but these costs are categorized as start-up phase as these revisions would not be expected to continue in future years
Development of monitoring systems	Concept and system designed by the World Bank, including both offline and web-based management information systems
Printing of JHIC	Printing of JHIC by private printing firm contracted by KMPDC
**Implementation phase (November 2016 to December 2017)**	
Inspection	Health facility inspections conducted by 8 full-time inspectors supervised by MoH coordinator with support from World Bank team
Printing of score cards	Printing of scorecards by a private firm contracted by KMPDC
Closure visits	Closure visits to facilities by MoH coordinator accompanied by county officials. Involved posting a closure scorecard and notifying in-charge of the decision and process to obtain a license. Closure visits happened on average 70-days after the closure report from the inspection [15]
System maintenance	Routine maintenance of online monitoring system and SMS verification system for checking scorecard authenticity by the World Bank
General management	General JHI management, including meetings to discuss issues arising in the field and amend inspection protocols and procedures; 5-day refresher training of inspectors in Meru led by ITEG and World Bank team; monitoring and quality control activities including feedback to inspectors

The development phase encompassed all activities up to the point when an official decision was made to go ahead with the JHI pilot in December 2014. It was hard to choose a specific start date for the development phase as discussions about potential regulatory reforms had been ongoing for many years, including development of an earlier checklist. We chose October 2013 as the starting point as this marked the “Windsor Agreement” when a fresh mandate was given for the full revision of the inspection process. The development phase involved the design and piloting of the JHIC and associated scoring system, consultation and consensus building among stakeholders, and the JHIC legal gazettement.

The start-up phase started after the official go ahead was given (January 2015), and included all activities needed to prepare for implementation. This comprised national and county launch meetings; development of the implementation manual, scorecards, scorecard authentication system, patient leaflets, electronic tablet-based JHIC and monitoring system; printing of JHICs; training of trainers and inspectors; and facility mapping.

The implementation phase involved all activities that would need to be repeated on an annual basis: the inspections themselves, closure visits, routine maintenance of the monitoring and SMS verification systems, scorecard printing, and general management. This was based on costs incurred between November 2016 and December 2017.

The KePSIE RCT encompassed all private and public health facilities in the three counties. Each county was divided into “health markets”, defined as a cluster of facilities where no facility was more than four kilometers away from the geometric centre of the cluster. A total of 273 health markets were randomly assigned to one of three arms: in arms 1 and 2 all public and private facilities were inspected at least once annually, following the JHI protocol. In addition, in arm 2, inspection performance scorecards were publicly displayed at the facility. Arm 3 continued with normal practice (which effectively meant no facilities were inspected in 2017).

Implementation of the JHI pilot covered 856 facilities in the intervention arms and involved 2,523 visits to these facilities for attempted inspection or closure (Table 4). These visits included 1,670 successfully completed inspections, 468 visits that did not lead to a completed inspection as, for example, the facility was closed, and 385 visits by the MoH Coordinator and county teams to enforce closure of facilities and departments. A typical inspection visit involved verification of the facility licenses, an interview with staff, and an audit of structural measures of patient safety such as protocols, infrastructure, equipment, and supplies. Inspections varied in length from a few minutes if the licenses were not valid, to 2 to 3 h for a primary health facility, or potentially a full day for a hospital.

**Table 4:** JHI pilot inspection and closure visits conducted

**Activities**	**No**
Facilities randomized into intervention arms	856
Completed inspections (1)	1670
Visits that did not end in completed inspections (2)	468
**Total inspection visits (1 + 2)**	**2138**
Visits for closures (3)	385
**Total visits to health facilities (1 + 2 + 3)**	**2523**

Source: JHI management information system

### Costing the JHI Pilot

The full economic costs of developing and implementing the JHI pilot were calculated, in line with the “Reference Case for Estimating the Costs of Global Health Services and Interventions” [16]. We adopted a broad provider perspective, including all costs incurred by agencies supporting the pilot (being the MoH, regulatory agencies, county governments, and the World Bank Group). Costs associated with research activities for the KePSIE evaluation were excluded, mainly comprising World Bank staff time and travel costs for RCT-related data collection and analysis. We did not include the costs of compliance incurred by health facilities, for example in obtaining licenses or upgrading the facility, or any costs to households.

The costing covered just over four years, from October 2013 to December 2017, divided into the three phases described above. Data collection began with an initial meeting with staff with leading roles in JHI to document its “production process” i.e., phases, activities in each phase, and resources used in each phase. Cost data were collected retrospectively using a mix of bottom-up and top-down approaches, depending on the resource. Sources for costing data comprised JHI implementation documents, financial records from the World Bank and KMPDC, and a series of seven key informant interviews with World Bank, MoH and KMPDC staff between January and December 2019.

For each resource used we identified reported expenditure or estimated this using the market price and quantity used (Additional file 1). The share of time spent by World Bank and MoH staff on each JHI activity was estimated through discussion with the core team and/or individuals concerned; World Bank staff were asked to estimate the share of time spent on implementation (as opposed to research activities), and to allocate their international travel costs on this basis. Office costs were based on typical commercial property rents. Other recurrent costs were based on reported expenditure e.g., inspector training, JHIC printing, contract for developing monitoring system etc. Capital costs were annualized using a discount rate of 3%. We estimated a useful life of 5 years for office equipment (laptops, modems, tablets, printers, furniture), and 8 years for vehicles. All prices were adjusted to 2017 prices using the Kenya GDP deflator [17] and converted to USD using an average annual exchange rate [18].

To derive typical annual economic costs of the JHI pilot, development and start-up phase costs were annualized over a useful life of 20 years and 7 years respectively, at a 3% discount rate, and summed with implementation phase costs for 2017. A useful life of 20 years for the development phase was chosen as the legal parameters of facility inspection are rarely changed. For the start-up phase the useful life was set at 7 years as an estimate of a reasonable time for specific approaches to implementation to be maintained. Annual economic unit costs were calculated per facility covered, per facility visit, and per completed inspection.

### Estimating the cost of national scale-up

We also modelled the costs of scaling-up the JHI model to all 47 counties in Kenya. A detailed description of how the costs for each resource were adjusted to national scale is provided in Additional file 2. Broadly, the costs of national-level activities such as JHIC development, training of trainers, and development of the monitoring system were assumed to be fixed, while other costs were estimated by scaling up JHI pilot costs based on number of counties, number of inspectors or number of facilities as appropriate. Three main changes were assumed to the JHI design to adapt it to national scale:

- Adapting the management structure to replace the MoH and World Bank staff involved in the pilot with 10% of the time of the recently formed Kenya Health Professionals Oversight Authority (KHPOA); a full-time National MoH Coordinator (100%); and eight full-time Regional MoH Coordinators (each covering approximately 6 counties).
- Increasing the number of inspectors from 8 to 147 (i.e., just over 3 per county) in line with current scale-up plans, with inspectors to be employed by MoH.
- Converting all World Bank salary costs to Kenya Government rates, and excluding international travel costs, based on the rationale that the original JHI pilot was novel and required additional international technical assistance, but for Kenyan scale up (or implementation in another country), locally employed staff would adapt the existing model.

To calculate unit costs for scale-up the total number of inspections was estimated by scaling up the 1,670 completed inspections during the JHI pilot pro rata, based on the ratio of the total number of health facilities nationwide to the number in the pilot treatment arms (12,438/856). We note that national scale-up in Kenya will not require a repeat of most development phase activities, or of some start-up phase activities, such as the development / piloting of the implementation manual, scorecards, SMS verification system, e-JHIC, and monitoring systems, though some similar activities may be conducted to make adaptations for scale-up. For completeness, and for relevance to other countries considering similar reforms, we report the scale-up costs both including and excluding these activities.

### Comparator – Pre-JHI standard inspections

As noted above, health facility inspection was taking place to some extent in Kenya pre-JHI. We therefore compared the costs of JHI scale-up to these earlier “standard inspections”. It was not meaningful to compare the pilot costs to standard inspections as in any one year it was quite probable that none of the 3 pilot counties would have been included in standard inspections. There was considerable variation in the standard inspections year on year, depending on resource availability and competing priorities. However, they were typically organized 2 or 3 times a year, with a team of regulatory staff from different agencies travelling together by car to a single region. Typically, just over 700 facilities were visited in one round of inspection over 5 working days. The KMPDC organized and oversaw inspection implementation. The standard inspections did not involve any joint checklist, monitoring system, training, manual, scorecards, or separate closure visits.

Standard inspection costs were estimated based on their implementation costs for the period November 2015 to November 2016. We did not use standard inspection costs from non-pilot counties in 2017, as standard inspections were particularly limited that year because stakeholders’ attention was focused on the pilot. Expenditure data were primarily obtained from KMPDC.

### Sensitivity analysis

One-way sensitivity analysis was conducted to explore the impact of varying key uncertain parameters on the unit costs of the JHI pilot and the national scale-up. Specifically, we varied the useful life of the development and start-up phases as these are inherently difficult to predict, and staff time allocated to JHI implementation, as staff time is the most substantial cost category and allocations of staff time are based on estimates by individuals and may be subject to recall bias. We also varied the number of inspectors to be deployed per county during national scale up.

## Results

### Cost of JHI pilot

The total economic costs of the JHI pilot were USD 1,125,600 (2017 USD) (Table 5). Of this total, the development phase accounted for 19%, the start-up phase 43%, and the implementation phase 38%. Individual activities accounting for the highest cost shares were inspections (29%), training of trainers and inspectors (21%), and JHIC development (10%). The high training costs of USD 237,185 reflected the training of 20 potential inspectors to allow for selection of the best performers, the high salary costs of staff involved in training facilitation, particularly those funded by the World Bank (32% of training costs), contracts for development of the training manual and facilitation of training of trainers (22%), and allowances for trainers and trainees (12%).

**Table 5:** Economic costs of JHI pilot and national scale-up by activity (2017 USD)

	**JHI pilot**			**National scale-up**		
	**USD**	**% of phase**	**% of total**	**USD**	**% of phase**	**% of total**
**Development phase**						
Development of JHIC	114,951	54%	10%	95,237	71%	2%
Consensus building	73,254	34%	7%	13,914	10%	0.3%
JHIC Gazettement	25,606	12%	2%	25,606	19%	1%
**Total development phase**	**213,811**	**100%**	**19%**	**134,758**	**100%**	**3%**
**Start-up phase**						
Implementation preparation	47,019	10%	4%	70,963	6%	1%
Development of implementation manual	35,941	7%	3%	15,574	1%	0.3%
Training & recruitment of inspectors	237,185	49%	21%	609,719	55%	13%
Preparation of materials	26,114	5%	2%	32,489	3%	1%
Facility mapping	24,457	5%	2%	87,628	8%	2%
Development of e-JHIC	23,497	5%	2%	16,758	2%	0.3%
Development of monitoring systems	82,133	17%	7%	76,718	7%	2%
Printing of JHIC	8,319	2%	1%	195,500	18%	4%
**Total start-up phase**	**484,665**	**100%**	**43%**	**1,105,350**	**100%**	**23%**
**Implementation phase**						
Inspections	328,986	77%	29%	3,178,690	89%	66%
Closure visits	35,450	8%	3%	41,254	1%	0.9%
Printing of scorecards	2,520	1%	0.2%	118,459	3%	2%
System maintenance	52,366	12%	5%	222,831	6%	5%
General management	7,801	2%	1%	22,385	1%	0.5%
**Total implementation phase**	**427,124**	**100%**	**38%**	**3,583,620**	**100%**	**74%**
**Grand total economic costs**	**1,125,600**	**-**	**100%**	**4,823,728**	**-**	**100%**
**Annualized costs:**						
Annualized development costs	14,371	-	3%	9,058	-	0.2%
Annualized start-up costs	77,792	-	15%	177,416	-	5%
Annual implementation costs	427,124	-	82%	3,583,620	-	95%
**Total annualized economic costs**	**519,287**	**-**	**100%**	**3,770,093**	**-**	**100%**

**Fig. 1:**
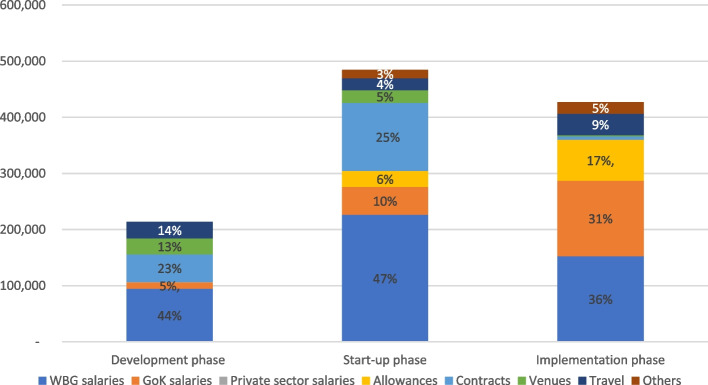
Economic costs of JHI pilot by cost category (2017 USD). Notes: WBG—World Bank Group GoK—Government of Kenya

With reference to cost categories, World Bank Group salaries comprised the largest share of total economic costs in each of the three phases, 44% in the development phase, 47% in the start-up phase, and 36% in the implementation phase (Fig. 1). Contracts with private firms (e.g., for JHIC development, legal advice, facilitation of training, scorecard design, and monitoring system design) also consumed a significant share of costs in the development and startup phases at 23% and 25% respectively. In the implementation phase Government salaries accounted for 31% of costs, and payment of daily subsistence allowances and top-up stipends to inspectors and the MoH coordinator for 17%. Economic costs by funder are provided in Additional file 3.

The annualized costs of the development and startup phases were USD 14,371 and USD 77,792 respectively, with the annual costs of routine implementation being USD 427,124 (Table 5). Summing these costs gave a total economic cost of a typical year of the JHI pilot of USD 519,287 (development phase 3%, start-up phase 15%, implementation phase 82%). This is equivalent to USD 607 per facility covered, USD 206 per facility visit (for inspection or closure) and USD 311 per inspection completed (Table 6). The largest share of the annualized costs were accounted for by World Bank Group salaries (38%), followed by Kenyan Government salaries (28%), and staff allowances (15%) (Additional file 4).

**Table 6:** Sensitivity analysis of annualized economic unit costs of JHI pilot and scale-up (2017 USD)

	**JHI pilot unit costs**		**National scale-up unit costs**	
	**Per facility visit**	**Per inspection completed**	**Per facility visit**	**Per inspection completed**
**Base case**	206	311	103	155
**Sensitivity analysis**				
**Annualization of development phase (base case 20 years)**				
10 years	210	317	103	156
30 years	204	309	103	155
**Annualization of start-up phase (base case 7 years)**				
5 years	217	328	105	158
10 years	198	298	102	153
**Staff time allocated to JHI activities**				
+ 25%	228	345	116	176
-25%	180	271	89	135
**Number of inspectors during the national scale up (base case 147 inspectors)**				
94 (2 inspectors per county)	-	-	72	109
188 (4 inspectors per county)	-	-	115	173

### Costs of national level scale-up

The total economic costs of national scale-up were estimated at USD 4,823,728 (Table 5), with 3% accounted for by the development phase, 23% by the start-up phase, and 74% by the implementation phase. As with the pilot, inspection and training were the activities accounting for the largest shares of total economic costs at 66% and 13% respectively. The annualized economic costs were estimated at USD 3,770,093 (0.2% development phase, 5% start-up phase, 95% implementation phase). Scaling up inspection activity from the pilot in line with 12,438 facilities nationwide leads to an estimated 36,660 facility visits, and 24,266 completed inspections. This translates to economic unit costs of scale-up of USD 103 per facility visit and USD 155 per inspection completed (Table 6).

If it was not necessary for the Government to repeat the development phase or several activities within the start-up phase (development / piloting of the implementation manual, scorecards, SMS verification system, e-JHIC, and monitoring systems), the total economic costs would fall to USD 4,547,430, and annualized economic costs to USD 3,720,217 giving an annualized economic unit cost of USD 102 per facility visit and USD 154 per inspection completed.

By way of comparison, the total economic costs of the standard inspections prior to JHI were estimated at USD 86,997, equivalent to USD 113 per facility inspected. Of these costs, USD 53,417 (61%) was spent in payment of daily subsistence allowances (Additional file 5).

### Sensitivity analysis

The sensitivity of the pilot and scale-up unit costs to variation in key parameters is shown in Table 6. The unit costs were relatively robust to reasonable ranges for the useful life of the development and start-up phases. Results were somewhat more sensitive to varying the staff time allocated to the JHI. For the pilot we varied ± 25% for all staff not full-time on JHI implementation (range of USD 271–345 per inspection completed). For the scale-up we varied all staff costs ± 25% as all staff time was estimated (range of USD 135–176 per inspection completed).

## Discussion

While there are many possible strategies for improving quality of care in health facilities, compliance with government regulation is considered a fundamental requirement for achieving minimum quality and safety standards. In many LMIC settings such regulation is highly inadequate [6], leading to concerning results. For example, in the three counties prior to JHI introduction, 97% of Kenyan facilities failed to meet minimum patient safety standards (67% categorized as minimally compliant and 30% partially compliant) [10]. Improving the frequency and effectiveness of facility inspection is therefore an important priority. The JHI pilot represented a set of important innovations in this area, in terms of the comprehensive checklist design, electronic data entry, public scorecards, and risk-based and responsive elements of the regulatory protocol. The pilot was successful in improving regulatory compliance by 15% in one year [11]. The Kenyan government is now in the process of scaling up an adapted version of the JHI pilot nationwide. Understanding the cost implications is therefore essential to assess the financial sustainability of such enhanced inspections and their value for money. As far as we are aware, this is the first study in sub-Saharan Africa to quantify the costs associated with either the previous standard facility inspections, or those following a JHI model.

We found that the total economic cost of developing and implementing the JHI pilot for one year in two thirds of facilities (the two intervention arms) in three counties was about USD 1.1 million, equivalent to an annualized economic cost of USD 519,287, and a unit cost per health visit and inspection completed of USD 206 and USD 311 respectively. Predicting the costs of national scale-up is obviously much more uncertain, but based on reasonable scaling assumptions, we estimated that development and implementation at a similar intensity for one year would have an economic cost of about USD 4.8 million, equivalent to an annualized economic cost of USD 3.8 million, and a unit cost per inspection completed of USD 155. This represents a substantial increase in total expenditure on inspections from standard practice prior to JHI. The cost per standard inspection at USD 113 was about a third of the annualized economic unit cost of the JHI pilot but of a roughly similar order of magnitude to the cost for national scale-up. However, the total economic cost of standard inspections was only 2.5% of the estimated total annualized economic cost of JHI scale up, reflecting the sporadic and patchy nature of standard inspections, their exclusion of public facilities, the less intensive nature of inspection process, systems and protocols, and the lack of follow up visits for facility closure. To assess affordability, we estimated the total costs of the implementation phase only for national scale-up to be USD 3.6 million, equivalent to 0.4% of government expenditure on health (USD 830 million in 2017 [19]).

It is important to highlight the sizable costs of the development and start-up phases of the JHI process – amounting to USD 213,811 and USD 484,665 respectively in the pilot – as the level of attention given to technical design, inspector training, and consensus building activities was seen as central in the pilot’s success [20]. Vassall et al. stress the importance of including such costs, which they refer to as the “cost of supporting change” [16]. Other countries wanting to implement similar reforms may be able to draw heavily on the JHI materials and therefore substantially reduce these costs, though adaptation to local context, rigorous training, and ensuring buy-in from a wide range of stakeholders will still be required.

Other strengths of the costing include the operational “real world” nature of inspection implementation under JHI, and the full year duration of the implementation phase, allowing us to capture any seasonal cost variations, for example due to public holidays or weather. However, it is important to consider how typical the pilot costs are likely to be of future implementation. First, 2017 was an eventful year in Kenya with two health worker strikes lasting several months and two general elections. This hampered inspection productivity, without reducing most costs, indicating that unit costs might have been lower in other years. Secondly, the JHI pilot benefited from strong support from the World Bank – both from senior staff from Nairobi and Washington DC, and other Kenya-based staff. This is clearly evident in the 42% of total economic costs accounted for by World Bank staff salaries and 4% by travel costs for DC staff. When calculating scale-up costs we have assumed that these staff would be replaced with MoH employees at appropriate managerial grades, which substantially reduces staff costs, though the potential impact on JHI activity levels and quality is hard to predict, a challenge shared with other costings of health system interventions [21]. Thirdly, as the pilot was implemented as part of an RCT, we have aimed to exclude any costs due to this evaluation, though the difference between ‘research’ and ‘implementation’ costs can be hard to define [16] especially as several World Bank staff were involved in both. The RCT design could also have influenced resource use due to the randomization by market Center, which may have reduced efficiency as inspectors had to travel further to reach a given number of facilities in the intervention arms. Where JHI inspections are implemented throughout a county, use of additional strategies such as mass media awareness campaigns could potentially enhance compliance.

We were only able to cost the first year of pilot implementation. One might expect resource use to change in subsequent years if for example, fewer inspections were required over time, as more facilities obtain valid licenses and improve other elements of compliance, thus reducing the need for closure visits and increasing the time to re-inspection (Table 2). However, this may take time to emerge, given that facility turnover is quite high and their numbers are growing: over a 3-year period, of all facilities operating in the RCT control areas, 11% closed down, and 37% new facilities opened [11]. Also, the inspectors were not able to meet specified timelines for re-inspections and closures during the pilot, so would likely need to maintain current capacity just to do more visits on time. Finally, the JHI model is likely to change as it is adapted for national scale-up. For example, the government does not plan to include scorecards or enforce closures during the initial years: removing all activities related to scorecards and closures would reduce the total economic costs for national scale up by 3% and 1% respectively. There is also the question of how generalizable the pilot costs will be to other counties. The three pilot counties were selected to exhibit variation in key characteristics (Table 1), but they all had a relatively high number of facilities, compared with the national mean of 265 per county, though much lower facility density than the metropolitan centres of Nairobi, Mombasa, and Kisumu. For all these reasons, it will be important to confirm the modelled scale-up costs after national roll out in Kenya.

Costs were calculated from a regulator / provider perspective, including all costs incurred by the MoH, regulatory agencies, county governments, and the World Bank. We felt this was appropriate as regulators will be interested in their resource use and the value for money of their own activities. To some degree the JHI will also have led to increased revenues for regulatory agencies as a result of greater compliance with facility licenses, though these data were not available for inclusion in this analysis. It is also important to consider whether there are broader societal costs. Costs to households as a direct result of inspections were likely to be low, limited to longer waiting times during inspections. It is possible that some facility closures led to additional travel costs for households. However, the number of inactive facilities in the year following the pilot was not found to be dramatically higher in the intervention arms than in control areas [11]. More importantly, we have not included costs to health facilities of their time during inspections, of complying with the regulations in terms of obtaining licenses or upgrading the facility, or of lost revenues due to closures. We were not able to obtain quantitative data on such costs but did explore this issue during qualitative interviews as part of the broader study. While some said they made required improvements using their existing resources, many others said they incurred additional costs, with amounts highly variable, typically ranging from a few hundred to a few thousand dollars, though a few gave much higher estimates. Renewing or obtaining new licenses was said to cost around USD 20 and USD 150 respectively. A few facilities described lost income from closures, for example, USD 150 lost per month for closure of a lab, and USD 600 for the whole facility. However, many facilities also noted that payment for bribes to inspectors was almost unheard of under JHI, whereas this had been prevalent under previous inspection regimes.

It is difficult to identify appropriate studies to compare these results to, as we are not aware of other regulatory costings in similar contexts. There is a general lack of cost and cost-effectiveness data on any interventions to improve health facility performance, such as regulation, social franchising, accreditation or other quality improvement strategies [22]. A possible comparator is costings of performance-based financing, which have estimated an annual implementation cost (excluding design phase) per facility covered of USD 8,920 [23], USD 28,397 [24] and USD 108,631 [21] (all converted to 2017 USD), compared to an equivalent of USD 539 per facility for the JHI pilot (implementation phase only). However, the interventions are very different as the pay for performance involves substantial incentive payments to facilities.

The key question is whether policy makers consider the costs of JHI inspections good value for money in comparison to alternative uses of their constrained resources. One could argue that given the importance of patient safety, and the government’s clear stewardship role in this regard, that JHI could be seen as good value for money, if it leads to sustained increases in regulatory compliance. To fully assess the cost-effectiveness of the inspections, one would need to know whether such increases in compliance translated to improvements in patient safety and quality of care, and ultimately final health outcomes, and this is difficult to predict, as there is often poor correlation between the structural quality measures included in inspection checklists and clinical quality of care [25].

## Conclusion

This study provides robust estimates of the cost of rigorous health facility inspection. The JHI was estimated to cost USD 311 per inspection completed during the pilot phase, and USD 155 in a modelled national scale-up. National implementation would involve a major increase in resources for regulation compared to previous standard sporadic inspections but appears potentially affordable compared to total government health expenditure and could represent good value for money if it leads to sustained increases in regulatory compliance in the public and private sector. These findings will serve as an important benchmark for future studies, and a key input into future cost-effectiveness analyses of facility regulation.

## Supplementary Information

**Additional file 1. **Methods for calculating cost of each resource used in JHI pilot.

**Additional file 2. **Methods for scaling up JHI pilot costs to the national level.

**Additional file 3. **Economic and financial costs of JHI pilot by phase and funder (2017 USD).

**Additional file 4. **Breakdown of annualized cost of JHI pilot by cost category (2017 USD).

**Additional file 5. **Breakdown of costs of standard inspections by cost category (2017 USD).

## Data Availability

The datasets generated during and/or analyzed during the current study are not publicly available as they include confidential financial but are available from the corresponding author on reasonable request.
