# Supplementary material for: How much does effective health facility inspection cost? An analysis of the economic costs of Kenya’s Joint Health Inspection innovations
Source: BMC Health Serv Res. 2022 Nov 14;22:1351. doi: 10.1186/s12913-022-08727-3 (PMC9664811; doi:10.1186/s12913-022-08727-3)
Supplement: Supplementary file 1 — Additional file 1. Methods for calculating cost of each resource used in JHI pilot. [file 12913_2022_8727_MOESM1_ESM.docx]

**Supplementary data tables**

**Additional File 1. Methods for calculating cost of each resource used in JHI pilot.**

| **Activity phase** | **Activity** | **Inputs** | **Costing methods** |
| --- | --- | --- | --- |
| **Development phase** | Development of JHIC | Staff time – MoH staff, World Bank Group (WBG) staff, ITEG | - Share of salary for the days dedicated^^[[1]](#footnote-1)^^ to this activity over total working days in a year (216) for MOH staff and ITEG members. Salaries for MoH staff were obtained from Government circulars for civil servants, and staff allowances from the Salaries and Remuneration Commission. - Salary expenditures reported by WBG for WBG staff. |
|  |  | Venue – World Bank (WB) offices and 2 hotels | Amount paid to the 2 hotels used (Windsor and Safari Park); Share of monthly rent of WB offices in relation to the number of days the offices were used |
|  |  | Contracts develop the checklist | Full amount paid for the contract |
|  | Consensus building | Staff time – MoH, WBG staff, and KTF principals | - Share of salary for the days dedicated to this activity over total working days in a year (216) for MOH staff and KTF principals. Source for salaries and allowances as above. - Salary expenditures reported by WBG for WBG staff |
|  | Gazetting JHIC | Consultant | Amount paid as reported in the expenditure records from World Bank |
|  |  |  |  |
| **Startup phase** | Implementation preparation: Launch of JHIC, kick-off meetings in counties, and distribution of JHIC to CHMT and facility managers | Vehicles | Share of annualized costs of a vehicle by the KMs covered during this activity and converted to months to reflect the equivalent number of months it was in use. |
|  |  | Staff time – MOH staff, WBG staff, ITEG members, regulatory agencies staff, | - Share of salary for the days dedicated to this activity over total working days in a year (216) for MOH staff, regulatory agencies staff, and ITEG members. Source for salaries and allowances as above. - Salary expenditures reported by WBG for WBG staff. |
|  |  | Venue – Safari Park hotel for the launch of JHIC | As reported in the expenditure records |
|  | Development of implementation manual | Contract | Amount paid as reported in the expenditure records |
|  | Training and recruitment of inspectors | Staff time – MOH staff, WBG staff, inspectors, ITEG members, regulatory agency staff, | - Share of salary for the days dedicated to this activity over total working days in a year (216) for MOH staff inspectors, regulatory agencies staff, and ITEG members. Source for salaries and allowances as above. - Salary expenditures reported by WBG for WBG staff. |
|  |  | Venue | - For training venue (Lake Naivasha Country Club) – as reported in the expenditure records. - For recruitment of inspectors (KMPDC offices) – share of monthly rent by the number of days used. |
|  | Design & piloting of scorecards, design of SMS verification for score card | Contracts | Contracts paid as reported in the expenditure records from WBG |
|  | Facility mapping | Staff time – MOH staff, WBG staff | - Share of salary for the days dedicated to this activity over total working days in a year (216) for MOH staff. Source for salaries and allowances as above. - Salary expenditures reported by WBG for WBG staff. |
|  |  | Transport | As reported in the expenditure records from WBG |
|  | Development of e-JHIC | Contract | Contracts paid as reported in the expenditure records from WBG |
|  | Development of web-based and offline monitoring systems | Contract | Contracts paid as reported in the expenditure records from WBG |
|  | Printing JHIC | Contract | Contracts paid from expenditure records from KMPDC |
|  |  |  |  |
| **Implementation phase** | Inspection | Staff time – MOH staff, WBG staff, ITEG members, inspectors, and drivers | - Share of salary for the days dedicated to this activity over total working days in a year (216) for MOH staff inspectors, and ITEG members. Source for salaries and allowances as above. - Salary expenditures reported by WBG for WBG staff. |
|  |  | Vehicles | - Share of annualized costs of a vehicle by the KMs covered during this activity and converted to months to reflect the equivalent number of months it was in use. - The annualized capital costs of 4-wheel drive vehicles per activity were based on the estimated share of time (in months) the three vehicles were used for each activity, replacement costs from Toyota Kenya, and a useful life of 8 years |
|  |  | Equipment – Laptop, printer, furniture, modems, tablets | Annualized capitals costs over an estimated useful life of 5 years for each equipment at 3% discount rate multiplied by quantities used |
|  |  | - Scorecard printing - Internet connectivity - Server storage - SMS solution for score card verification - Communication expenses - DSA for inspectors - Inspection field transport (fuel) - Inspectors’ materials - DHL to transport JHIC, scorecards and patient information leaflets to counties | As reported in the expenditure records from KMPDC. |
|  |  | Venue - county offices | Share of imputed monthly rent by the number of days used |
|  | Closure | Staff time – MOH staff, WBG staff, inspectors | - Share of salary for the days dedicated to this activity over total working days in a year (216) for MOH staff inspectors. Source for salaries and allowances as above. - Salary expenditures reported by WBG for WBG staff. |
|  | General management: governance meetings, refresher training, mid-implementation review, monitoring and quality checks | Staff time – MOH staff, senior WBG staff, ITEG members, and regulatory agencies staff | - Share of salary for the days dedicated to this activity over total working days in a year (216) for MOH staff ITEG members, regulatory agencies staff. Source for salaries and allowances as above. - Salary expenditures reported by WBG for senior WBG staff. |
|  |  | Venue – Golf Hotel Kakamega for inspector refresher trainings | As reported in the expenditure records from KMPDC |
|  | Maintenance of monitoring system & SMS verification system for scorecards | Contracts | Amount paid as reported in the expenditure records from KMPDC |

1. Days estimated through discussion with the core team and/or individuals concerned. [↑](#footnote-ref-1)
