# Supplementary material for: How much does effective health facility inspection cost? An analysis of the economic costs of Kenya’s Joint Health Inspection innovations
Source: BMC Health Serv Res. 2022 Nov 14;22:1351. doi: 10.1186/s12913-022-08727-3 (PMC9664811; doi:10.1186/s12913-022-08727-3)
Supplement: Supplementary file 2 — Additional file 2. Methods for scaling up JHI pilot costs to the national level. [file 12913_2022_8727_MOESM2_ESM.docx]

**Additional File 2. Methods for scaling up JHI pilot costs to the national level**

| **Phase** | **Activity** | **JHI pilot resources** | **Approach for estimating scale up costs** |
| --- | --- | --- | --- |
| **Development** | Development of JHIC | MoH staff | Fixed |
|  |  | World Bank staff |  |
|  |  | Inspector Training Expert Training Group (ITEG) members |  |
|  |  | Venues |  |
|  |  | Contract for developing JHIC |  |
|  | Consensus building | MoH officials | Fixed |
|  |  | World Bank staff |  |
|  |  | KePSIE Task Force principals |  |
|  | Gazette process | Consultant | Fixed |
| **Start-up** | Implementation preparation: Launch of JHIC, kick-off meetings in counties, and distribution of JHIC to CHMT and facility managers | Vehicles | Assumed each county would have one vehicle thus scaled up by (47/3) |
|  |  | MoH staff | Replaced with 8 MoH coordinators of job group P and a national coordinator |
|  |  | World Bank staff |  |
|  |  | ITEG members | Fixed |
|  |  | Regulatory agency staff | Fixed |
|  |  | Venues | Fixed |
|  |  | County kick-off meetings | Scaled up by number of counties (47/3) |
|  | Development of implementation manual | Contract | Fixed |
|  | Training and recruitment of inspectors | MoH staff | Replaced with 8 MoH coordinators of job group P and the national coordinator |
|  |  | World Bank staff |  |
|  |  | Inspector | Number increased from 20 trained in pilot to 147 |
|  |  | ITEG members (TOTs) | Fixed |
|  |  | Regulatory agency staff | Fixed |
|  |  | KMPDC offices | Fixed |
|  |  | Venues | By number of inspectors (147/20) |
|  | Design & piloting of scorecards, design of SMS verification for score card | Contracts | Fixed |
|  | Facility mapping | MoH staff | Replaced with 8 MoH coordinators of job group P and the national coordinator |
|  |  | World Bank Staff |  |
|  |  | Transport | Scaled up by county coverage (estimated by scaling up for control arm in pilot counties (3/2) and for number of counties (47/3) |
|  | Development of e-JHIC | Contract | Fixed |
|  | Development of web-based and offline monitoring systems | Contract | Fixed |
|  | Printing JHIC | Contract | Scaled up by county coverage (estimated by scaling up for control arm in pilot counties (3/2) and for number of counties (47/3) |
| **Implementation** | Inspection | Vehicles | By number of counties (47/3) |
|  |  | Equipment | By number of counties (47/3) |
|  |  | MoH staff | Replaced with 8 MoH coordinators of job group P and the national coordinator |
|  |  | World Bank staff |  |
|  |  | Inspectors | Number increased from 10 working at any time under JHI pilot to 147 |
|  |  | ITEG members | Fixed |
|  |  | Drivers | Scaled up by number of counties (47/3) |
|  |  | Scorecard printing | Scaled up by number of facilities (estimated by scaling up for 1 pilot arm with scorecards (3/1) and for number of counties (47/3) |
|  |  | Internet connectivity | Scaled up by number of facilities (estimated by scaling up for 1 pilot arm with scorecards (3/1) and for number of counties (47/3) |
|  |  | Server storage | Scaled up by number of facilities (estimated by scaling up for 1 pilot arm with scorecards (3/1) and for number of counties (47/3) |
|  |  | SMS Solution (scorecard verification system) | Scaled up by number of facilities (estimated by scaling up for 1 pilot arm with scorecards (3/1) and for number of counties (47/3) |
|  |  | Daily allowances (inspectors) | Scaled up by number of inspectors working at any time under JHI pilot to 147 currently undergoing training |
|  |  | Inspection field transport (Fuel) | Scaled up by county coverage (estimated by scaling up for control arm in pilot counties (3/2) and for number of counties (47/3) |
|  |  | Communication expenses |  |
|  |  | Inspector materials |  |
|  |  | DHL to transport JHIC, scorecards and patient information leaflets to counties | Scaled up by county coverage (estimated by scaling up for control arm in pilot counties (3/2) and for number of counties (47/3) |
|  |  | County offices | Scaled up by number of counties (47/3) |
|  | Closure visits | MoH staff | Replaced with 8 MoH coordinators of job group P and the national coordinator |
|  |  | World Bank staff |  |
|  |  | Inspectors | Number increased from 10 working at any time under JHI pilot to 147 |
|  | General management: governance meetings, refresher training, mid-implementation review, monitoring and quality checks | Vehicles | Scaled up by number of counties (47/3) |
|  |  | MoH and senior World Bank officials | Introduced KHPOA members, plus 8 MoH coordinators of job group P and the national coordinator |
|  |  | ITEG members | Fixed |
|  |  | Regulatory agency staff | Fixed plus addition of 2 administrative staff |
|  |  | Venue | fixed |
|  | Maintenance of monitoring system & SMS verification system for scorecards | Contract | Fixed |
