# Supplementary material for: How much does effective health facility inspection cost? An analysis of the economic costs of Kenya’s Joint Health Inspection innovations
Source: BMC Health Serv Res. 2022 Nov 14;22:1351. doi: 10.1186/s12913-022-08727-3 (PMC9664811; doi:10.1186/s12913-022-08727-3)
Supplement: Supplementary file 3 — Additional file 3. Economic and financial costs of JHI pilot by phase and funder (2017 USD). [file 12913_2022_8727_MOESM3_ESM.docx]

**Additional File 3. Economic and financial costs of JHI pilot by phase and funder (2017 USD)**

|  | **Development phase** | | **Start-up phase** | | **Implementation phase** | | **Total costs** | |
| --- | --- | --- | --- | --- | --- | --- | --- | --- |
|  | **USD** | **%** | **USD** | **%** | **USD** | **%** | **USD** | **%** |
| **Economic costs** |  |  |  |  |  |  |  |  |
| World Bank Group | 178,987 | 94 | 448,697 | 90 | 290,667 | 63 | 918,352 | 80 |
| Ministry of Health | 2,166 | 1 | 5,927 | 1 | 31,452 | 7 | 39,545 | 3 |
| County governments | 878 | 0.5 | 1,037 | 0.2 | 9,308 | 2 | 11,223 | 1 |
| Regulatory agencies | 7,906 | 4 | 43,740 | 9 | 129,839 | 28 | 181,485 | 16 |
| Private sector | 1,139 | 1 | - | 0 | - | 0 | 1,139 | 0.1 |
| **Total economic costs** | **191,076** | **100** | **499,401** | **100** | **461,265** | **100** | **1,151,744** | **100** |
