# Supplementary material for: How much does effective health facility inspection cost? An analysis of the economic costs of Kenya’s Joint Health Inspection innovations
Source: BMC Health Serv Res. 2022 Nov 14;22:1351. doi: 10.1186/s12913-022-08727-3 (PMC9664811; doi:10.1186/s12913-022-08727-3)
Supplement: Supplementary file 4 — Additional file 4. Breakdown of annualized cost of JHI pilot by cost category (2017 USD). [file 12913_2022_8727_MOESM4_ESM.docx]

**Additional File 4. Breakdown of annualized cost of JHI pilot by cost category (2017 USD)**

|  | **Annualized Dev costs** | **Annualized start-up costs** | **Annual Implementation costs** | **Total Annualized costs** | **% of total annualized costs** |
| --- | --- | --- | --- | --- | --- |
| World Bank Group salaries | 6,375 | 36,367 | 152,811 | **195,553** | **37.7%** |
| Government of Kenya salaries | 736 | 7,941 | 134,300 | **142,977** | **27.5%** |
| Private sector salaries | 77 | - | - | **77** | **0.01%** |
| Allowances | - | 4,549 | 73,182 | **77,730** | **15.0%** |
| Contracts | 3,299 | 19,523 | 5,773 | **28,594** | **5.5%** |
| Venues | 1,909 | 3,609 | 2,025 | **7,543** | **1.5%** |
| International travel for World Bank staff | 1,976 | 2,319 | 5,250 | **9,545** | **1.8%** |
| Local travel | - | 1,119 | 33,474 | **34,594** | **6.7%** |
| Other | - | 2,365 | 20,310 | **22,675** | **4.4%** |
| **Total costs** | **14,371** | **77,792** | **427,124** | **519,287** | **100%** |
