# Supplementary material for: How much does effective health facility inspection cost? An analysis of the economic costs of Kenya’s Joint Health Inspection innovations
Source: BMC Health Serv Res. 2022 Nov 14;22:1351. doi: 10.1186/s12913-022-08727-3 (PMC9664811; doi:10.1186/s12913-022-08727-3)
Supplement: Supplementary file 5 — Additional file 5. Breakdown of costs of standard inspections by cost category (2017 USD). [file 12913_2022_8727_MOESM5_ESM.docx]

**Additional File 5. Breakdown of costs of standard inspections by cost category (2017 USD)**

| **Cost category** | **USD** | **%** |
| --- | --- | --- |
| Salary | 19,753 | 23% |
| Allowances | 53,417 | 61% |
| Contracts | - | 0% |
| Venues | - | 0% |
| Travel | 12,865 | 15% |
| Other | 961 | 1% |
| **Total costs** | **86,997** | **100%** |
